# Gaze-contingent perceptually enabled interactions in the operating theatre

**DOI:** 10.1007/s11548-017-1580-y

**Published:** 2017-04-10

**Authors:** Alexandros A. Kogkas, Ara Darzi, George P. Mylonas

**Affiliations:** HARMS Lab, Department of Surgery and Cancer, Imperial College London, St Mary’s Hospital, 20 South Wharf Road, 3rd Floor Paterson Centre, London, W21PF UK

**Keywords:** 3D eye-tracking, Gaze contingent, Perceptually enabled interactions, SLAM, Smart operating theatre, Robot control

## Abstract

**Purpose:**

Improved surgical outcome and patient safety in the operating theatre are constant challenges. We hypothesise that a framework that collects and utilises information —especially perceptually enabled ones—from multiple sources, could help to meet the above goals. This paper presents some core functionalities of a wider low-cost framework under development that allows perceptually enabled interaction within the surgical environment.

**Methods:**

The synergy of wearable eye-tracking and advanced computer vision methodologies, such as SLAM, is exploited. As a demonstration of one of the framework’s possible functionalities, an articulated collaborative robotic arm and laser pointer is integrated and the set-up is used to project the surgeon’s fixation point in 3D space.

**Results:**

The implementation is evaluated over 60 fixations on predefined targets, with distances between the subject and the targets of 92–212 cm and between the robot and the targets of 42–193 cm. The median overall system error is currently 3.98 cm. Its real-time potential is also highlighted.

**Conclusions:**

The work presented here represents an introduction and preliminary experimental validation of core functionalities of a larger framework under development. The proposed framework is geared towards a safer and more efficient surgical theatre.

**Electronic supplementary material:**

The online version of this article (doi:10.1007/s11548-017-1580-y) contains supplementary material, which is available to authorized users.

## Introduction

The operating theatre is reportedly the environment where unintentional patient harm is most likely to happen [1]. Some of the most influential factors are related to *suboptimal communication among the staff*, *poor flow of information*, *staff workload* and *fatigue* and the *sterility of the operating theatre* [2]. While new technologies may add complexity to the surgical workflow, at the same time they provide new opportunities for the design of systems and approaches that can enhance patient safety and improve workflow and efficiency. A number of initiatives have assessed the state of the art in technological developments and identified key areas where future innovative solutions could be used to optimise the operating environment, such as *cognitive simulation, informatics, “smart” imaging, “smart” environments, ergonomics/human factors *and *group-based communication technologies* [3].

In the spirit of the *Internet of Things* (*IoT*) and the recent explosion of data-driven sciences, it is anticipated that equipment, surgical instruments, consumables and staff will be fully integrated and networked within a “smart” operating suite. This could happen in a number of ways, such as electronically, using computer vision, RFID markers or other technologies [4, 5]. Partially integrated operating suites are already being provided by companies, such as the Karl Storz’s $\hbox {OR1}^{\mathrm{TM}}$ [6], where components of the surgical environment (e.g. endoscopic devices, video/data sources, surgical table, ceiling lights) can be tailored to and by the user and can be controlled from a central location within the sterile area. Such operating suites, where a large amount of information can be made available through a unique integrated system, offer tremendous opportunities for implementing novel human–computer interfaces, context-aware systems, automated procedures and augmented visualisation features.

Moreover, a significant body of research has explored “perceptually enabled” interactions in the sterile environment using technologies like 3D cameras, voice commands or eye-tracking [7]. This way the surgeon can be kept in the loop of decision-making and task execution in a seamless way that is likely to help improving overall operational performance and reducing communication errors. For example, hand-gestures and a voice-driven robotic nurse introduced by Jacob et al. has been shown to reduce the number of movements without significantly affecting task execution time compared to collaboration with human nurses [8]. Eye-tracking methodologies in particular have the potential to provide a “third hand” and a seamless way to allow perceptually enabled interactions within the surgical environment. Previous work has demonstrated screen-based gaze control of surgical instruments [9]. In robotic [10] and conventional laparoscopic [11] surgical settings, screen-based collaborative eye-tracking of multiple collaborators was shown to significantly improve verbal and nonverbal communication, task understanding, cooperation, task efficiency and outcome.

Overall, the work presented here draws inspiration from the increasing utilisation of data from diverse sources in conjunction with advances in machine learning. It is also fundamentally driven by the need to keep the surgeon and his/her physical interactions with the environment tightly integrated into the decision-making process. As an introduction to a wider multi-sensor framework under development, core functionalities presented in this paper include: real-time free-viewing 3D fixation localisation, spatial reconstruction and modelling of the operating theatre, co-registration of an articulated collaborative robotic arm. One or more wearable eye-tracking devices can be used in combination with RGB-D cameras and advanced computer vision techniques. The ultimate goal is to develop functionalities, methodologies, open-source software and a low-cost generic hardware framework that can be adapted to any operating theatre with minor modifications and effort. The accuracy and real-time potential of the exemplar application presented here are assessed. To the authors’ knowledge, this is the first work combining computer vision and theatre-wide 3D gaze tracking for perceptually enabled interactions.

## Methodology

**Fig. 1:**
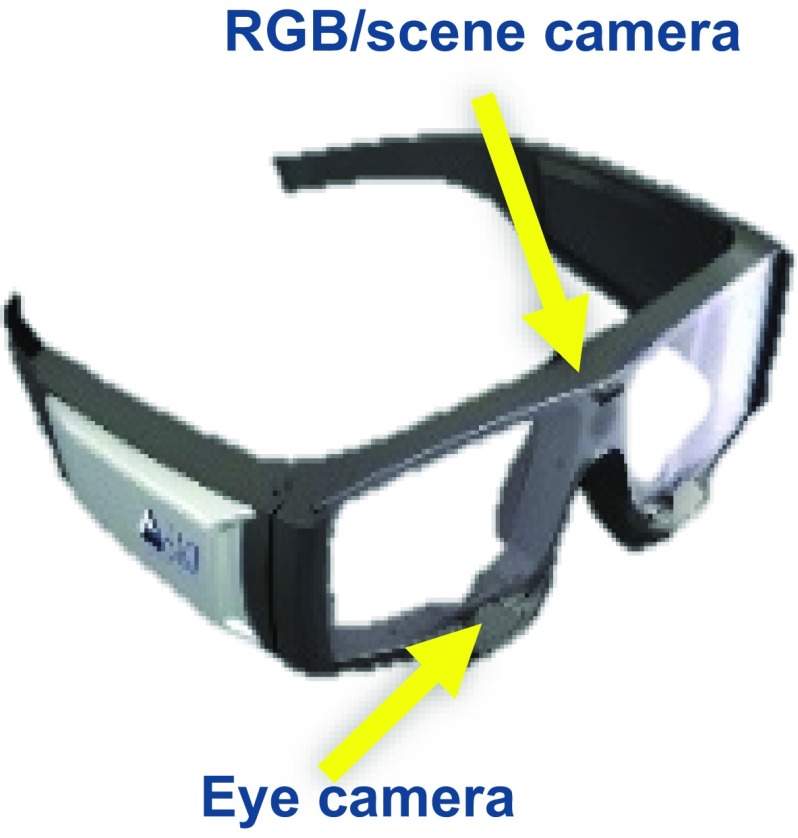
Wearable eye-trackers provide 2D gaze coordinates on the scene-camera frame of reference, which is equivalent to the head frame of reference

A core aspect of the envisaged framework is its capability to calculate and display the 3D fixation of one or more theatre attendants. An early version and preliminary evaluation of this functionality has been presented in [12] and [13]. A wearable eye-tracker and its integrated scene camera can be used to provide 2D gaze information and video of the scene in front of a user. After a short calibration routine, gaze direction vectors can be mapped to unique 2D gaze points on a virtual plane attached to the scene camera of the eye-tracker. This plane is fixed to and rotates with the user’s head (Fig. 1). Consequently, there is no direct quantitative correlation between 2D fixations and 3D positions of objects in space. To overcome this limitation, localisation of 3D fixations for this project is achieved through the combined use of conventional wearable eye-tracking and fixed in space RGB-D cameras for 3D reconstruction of the environment. A crucial functionality of the framework relies on the ability to provide an accurate estimate of one’s head pose (equivalent to the eye-tracker’s scene-camera pose) on a world coordinate system fixed with respect to the operating theatre. The pose is then used to map the 2D gaze information reported by the eye-tracker to a unique 3D fixation in the world frame of reference. A co-registered robot arm is used to point a laser pointer at the position of the resolved 3D fixation.

**Fig. 2:**
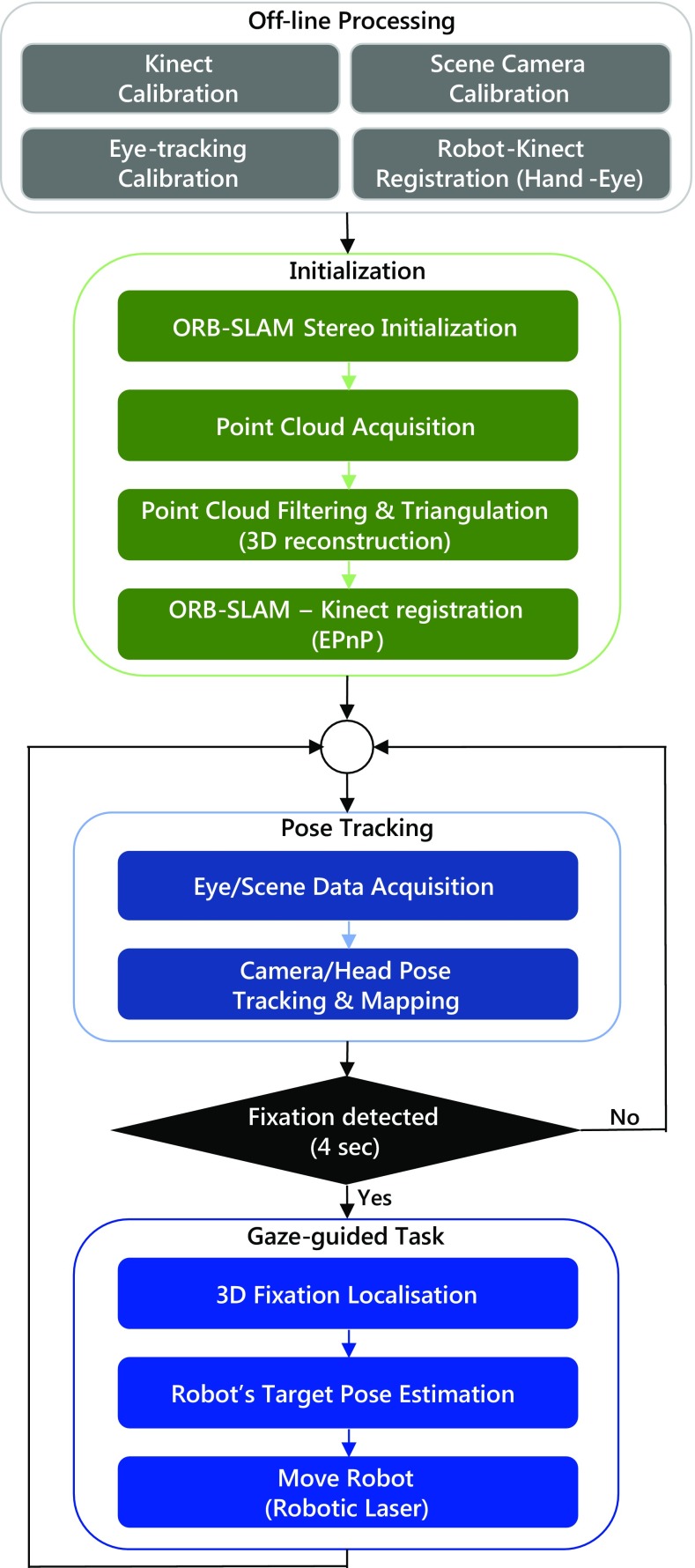
The implementation of the framework comprises four main phases: off-line processing, initialisation, pose tracking and the gaze-guided task

The implementation workflow of the functionality is shown in Fig. 2. During the off-line processing phase, the involved components are calibrated and co-registered. This requires determining the eye-tracker’s RGB scene-camera intrinsic parameters, the RGB-D camera colour-to-depth alignment and the eye-tracker’s gaze-to-scene video alignment. Additionally, the robot is registered to the world frame. During the initialisation phase, an initial mapping of the space and initial camera pose are estimated, a static 3D model is captured and processed, and the necessary registrations among coordinate systems are performed according to the application. The pose tracking phase involves real-time RGB scene video and gaze data recording by the eye-tracker. Then, the camera pose is tracked, and the map is updated simultaneously. Eventually, during the gaze-guided task phase, 3D fixation localisation is performed, based on mapping 2D gaze and camera/head pose information into 3D gaze information. Finally, the calculated tool centre point (TCP) position is transmitted to the robotic arm.

For the current implementation, all hardware components are connected to a single computer. C++ is the programming language used.

### Application workflow

During initialisation, the eye-tracking glasses and the RGB-D camera are calibrated. The local coordinate systems (robot, 3D map extracted by the eye-tracker monocular RGB scene camera) are registered to the RGB-D camera’s world coordinate system, and the laser pointer is aligned to the robot’s end-effector. A 3D model of the operating theatre is then extracted by the RGB-D camera, and the pose of the eye-tracker’s scene camera is estimated within it using the Simultaneous Localisation And Mapping (SLAM) technique [14]. Subsequently, the 2D fixations provided by the eye-tracking glasses are mapped to 3D world coordinates and provided to the robot. Finally, the appropriate robot pose is estimated in order to highlight the 3D fixation with the laser attached to its end-effector. Each of these steps is elaborated in the following sections.

**Fig. 3:**
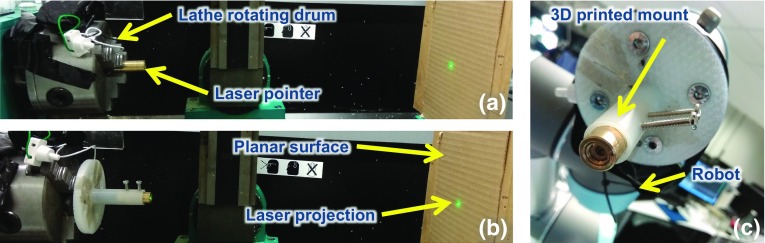
The laser module’s intrinsic calibration process

### Equipment

For eye-tracking the SMI Eye-tracking Glasses 2 Wireless (SensoMotoric Instruments GmbH) [15] are used. By tracking the position of the pupil and/or artificially generated features using near-infrared light sources and miniature cameras on the glass frame, the gaze direction of the user can be determined. The glasses operate as a fully mobile gaze-tracking device with 60-Hz sampling rate. An RGB scene camera with a resolution of 1280 $\times$ 960 pixels records an egocentric video at 24 frames per second. The field of view of the scene camera is $80^{\circ }$ (horizontal) and $60^{\circ }$ (vertical). Scene video and eye-tracking data are streamed in real time to a PC. The output of the system is a 2D gaze point on the image plane of the scene camera with a stated accuracy of $0.5^{\circ }$ of visual angle.

For RGB-D sensing, the Microsoft Kinect V2 is used for capturing depth and colour images concurrently. The Kinect uses an RGB camera with a resolution of 1920 $\times$ 1080 pixels at 30 Hz, an infrared emitter and an infrared camera with resolution of 512 $\times$ 424 at 30 Hz. It has 30-ms latency and 2- to 4-mm average depth accuracy error [16]. The field of view of the depth sensing is $70^{\circ }$ (horizontal) and $60^{\circ }$ (vertical), and it operates at distances between 50 cm and ${\sim }4.5\, \hbox {m}$. For depth estimation, the time-of-flight method is used [17].

The robot arm is a UR5 by Universal Robots. It is a collaborative robot providing 6 degrees of freedom, $\pm 360^{\circ }$ joint ranges, a reach radius of up to 850 mm and $\pm 0.1 \hbox {mm}$ repeatability. It weighs 18.4 kg and is capable of maximum 5-kg pay load.

To highlight the 3D fixation in the theatre, a green laser diode is attached on the robot’s end-effector using a 3D printed mount. As the laser beam is not exactly coincident with its z-axis, any alignment errors are corrected using a calibration step.

### Calibration

The accuracy of the calibration process is of paramount importance. Four types of calibrations are performed:

- Camera calibration for the eye-tracker’s RGB scene camera
- User-specific eye-tracking calibration of the eye-tracking glasses
- RGB-depth calibration for the Microsoft Kinect sensor
- Laser module to robot’s end-effector calibration

#### Eye-tracker’s RGB scene camera

The intrinsic and extrinsic camera parameters of the eye-tracker scene camera are calibrated using a chessboard.

#### Eye-tracking

Mapping eye fixations to specific points in the image plane of the video sequence, provided by the RGB/scene camera, requires a calibration procedure. During this procedure, users are asked to fixate on 3 predefined points in their field of view. Using the API provided by SMI [15], the parameters of a generic physiological 3D eye model are refined and the model is used to calculate the gaze vector. The model is a combination of shapes, light refraction and reflection properties of the different parts of the eyes. This process is not transparent and is dealt with internally by SMI algorithms.

#### Microsoft Kinect sensor

Although the RGB camera and the depth sensor of the Kinect are placed closely and capture similar planes, their slight spatial divergence may cause significant inaccuracies. The calibration process presented in [18] is used to align RGB and depth images.

#### Laser module

The laser module requires intrinsic calibration, as it produces an offset angle of ${\sim }0.8^{\circ }$, which is significant for projections over large distances. A mechanical offset calibration is used to align the laser module’s vector with the end-effector’s z-axis. The laser module is calibrated using a 3D printed component and screws. The pointer is first mounted on a lathe’s drum (Fig. 3a). By rotating the lathe and observing the laser projection on a planar surface, the projection centre and the diode angular offset direction are determined. Then, the pointer is mounted on a 3D printed base (mounted on the lathe) making sure the offset direction vector intercepts the line connecting the 2 screws (Fig. 3b). The screws are adjusted, while the lathe rotates, until the laser projects accurately to the projection centre on the planar surface. Finally, it is mounted on the robot’s end-effector (Fig. 3c).

### Registration

**Fig. 4:**
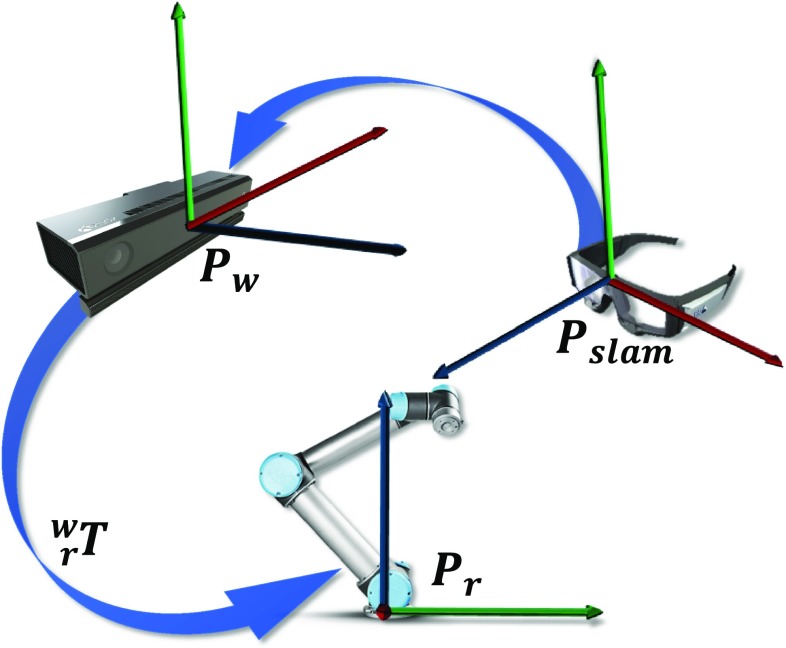
The transformations among the coordinate systems

In the proposed system, we use the Kinect’s coordinate system as the word frame of reference. To align multiple local coordinate systems to the global one, two main registrations are performed: a SLAM-to-Kinect and a Kinect-to-Robot registration (Fig. 4).

#### SLAM to Kinect

The head pose estimation relies on the localisation of a monocular camera within a local map, which is extracted during the initialisation of the SLAM algorithm [14]. Using a monocular camera for initialisation results to a scaled map, which is useful for tracking the camera pose in the 3D space. However, knowing the camera extrinsic parameters in relation to the world coordinates is desirable for two main reasons. First, 3D fixation localisation requires collision detection of the gaze vector with a triangulated point cloud. A sparse point cloud, as the SLAM map is, would result in inaccurate estimation of the 3D fixation. Second, the 3D fixation in world coordinates is necessary for gaze-guided tasks, such as robotic arm manipulation or object recognition.

The initial camera pose is estimated by the correspondences of the two initial keyframes. The first keyframe is the frame of reference of the map. The initial pose is used to triangulate the map and full global bundle adjustment to refine the initial map. For the registration, fiducial markers (for 2D–3D correspondences) and the EPnP algorithm [19] are employed to estimate the pose of the two first keyframes in the Kinect’s frame of reference. Defining the pose of the reference frame and the initial pose in the Kinect’s coordinates results to the extraction of the initial map in the world coordinate system.

#### Kinect to Robot

Accurate registration is necessary since minor inaccuracies can lead to significant deviation from the desired waypoints. The coordinate system of the robot is defined with respect to its base. The manipulation of a 6-axis robot involves calculation of 3D coordinates and rotation vectors, defining its pose. Therefore, Kinect-to-Robot registration is performed off-line using a chessboard pattern on the robot’s end-effector and the hand-eye calibration methodology presented in [20].

For every gaze-guided task, the TCP position is estimated in the world coordinate system. The robot receives the target pose in the robot coordinate system, calculated by:

1 $$\begin{aligned} \varvec{P}_{{\varvec{r}}} ={\varvec{{}_r^w T}}*{\varvec{P_w}} \end{aligned}$$

where ${\varvec{P_r}}$ is the TCP position in the robot coordinate system, ${\varvec{P_w}}$ is the TCP position in the world coordinate system, ${\varvec{{ }_r^w T}}$ is the transformation matrix from the world to the robot coordinate system obtained using the hand-eye calibration method [20].

### 3D spatial reconstruction

An essential part of the proposed framework is the real-time continuous spatial reconstruction of the theatre. The Microsoft Kinect V2 is employed to acquire the depth information of the scene as a depth image. Then, the depth image is converted into a point cloud using the *PCL library* [21]. While the point cloud contains all the necessary information about the scene, a triangulation process is required to convert it into a meshed surface for subsequent collision detection of the gaze vector with it, thus obtaining the 3D fixation.

Before triangulation, pre-processing of the point cloud is performed. At first we remove the outliers using statistical analysis techniques. Assuming the distribution of the points to their neighbours is *Gaussian*, all points with mean distance outside the interval defined by the mean distance of all points to all their neighbours, are removed. Then, the point cloud is downsampled into one-fifth of its original size, using a voxelised grid approach. A 3D voxel grid is created, and the points are limited to the centroids of each voxel. Eventually, a *Moving Least Squares* (MLS) surface reconstruction method is used to smooth and resample the noisy parts of the point cloud. This is achieved using higher-order polynomial interpolations between the surrounding data points to recreate missing parts of the surface.

Fast triangulation of unordered clouds by Marton et al. [22] is used. This method relies on maintaining a list of points from which the mesh can grow and extend until all possible points are connected. It can deal with unorganised points, coming from one or multiple scans, and having multiple connected parts. It performs optimally on smooth surfaces and areas with smooth transitions between different point densities. The triangulation is performed locally, by projecting the local neighbourhood of a point along the point’s normal, and connecting unconnected points.

A live and dynamically updated 3D model would introduce significant computational cost through real-time 3D updating and re-processing. For this reason, evaluation of the early implementation of the framework is based only on RGB and depth information acquired once, during the initialisation phase of building a static 3D model.

### Head pose estimation

The estimation of the head pose in the theatre is the most critical part in the proposed framework. There are several possible approaches to perform this task. Optical trackers or stereo cameras can be used to determine accurately the head pose in a world coordinate system. However, these would contradict our approach, which is based on a low-cost infrastructure, easy and generic deployment in several operating theatres and the avoidance of cumbersome equipment carried by the surgeon and staff. Instead, the synergy of advanced computer vision techniques and 3D spatial reconstruction is used to estimate the eye-tracker’s integrated scene-camera pose. SLAM [14] is a technique of building a map of an unknown environment by a mobile robot and estimating its pose within it. It consists of multiple phases, each of which can be computed in multiple ways: landmark extraction, data association, state estimation, state update and landmark update.

The ORB-SLAM method [23] is used with a monocular camera to estimate its pose in a 3D environment and map features of video frames. This method is robust to severe motion clutter, allows wide baseline loop closing and re-localisation and includes full automatic initialisation. The main tasks performed are: tracking, mapping, re-localisation and loop closing. Tracking refers to the estimation of the relative position of the camera to the scene objects in real time. Mapping refers to the construction of a 3D map of the environment in which the camera moves. Using a short video sequence, ORB-SLAM generates an initial map using ORB features and a homography assuming a planar scene, or a fundamental matrix assuming a nonplanar scene. Then, it performs builds/updates of the keyframe-based map and tracks the camera pose (extrinsic parameters) related to it. ORB-SLAM uses *bundle adjustment* for the map initialisation, local mapping and loop closing.

### 2D to 3D fixation localisation

The mapping of 2D fixations to 3D world coordinates consists of 3 main steps:

1. Classification of gaze into fixations and saccades (4-s dwell time threshold)
2. Calculation of the ray direction of the gaze vector.
3. Calculation of the intersection between the ray and the triangulated 3D space.

To estimate the ray direction vector, two points need to be calculated, the 2D fixation in world coordinates and the camera centre of projection. The ray is defined by the line connecting these two points. First, the 2D point ${\varvec{X_c}}$ is transformed in the camera coordinate system:

2 $$\begin{aligned} \varvec{X}_{\varvec{c}} =\varvec{K}^{-\mathbf{1}}{{\varvec{p}}}_{{\varvec{c}}} \end{aligned}$$

then the point is transformed in the world coordinate system:

3 $$\begin{aligned} {\varvec{X}_{{\varvec{w}}}} ={\varvec{R}^{-\mathbf{1}}\left( {{{\varvec{X}}}_{{\varvec{c}}} -{{\varvec{T}}}}\right) } \end{aligned}$$

and finally the centre of projection ${\varvec{C}_{\mathbf{op }}}$ is calculated:

4 $$\begin{aligned} {\varvec{C}_{\mathbf{op }}} ={-\varvec{R}^{-\mathbf{1}}{} \mathbf{T}} \end{aligned}$$

where $${\varvec{p}_{{\varvec{c}}}} ={[ \begin{array}{lll} {{\varvec{u}}}&{} {{\varvec{v}}}&{} \mathbf{1 }\\ \end{array} ]^{{{\varvec{T}}}}}$$ is the homogenous coordinates of the image point, ${\varvec{K}}$ is the matrix of intrinsic camera parameters and ${[\varvec{R} | \varvec{T}]}$ are the rotation and translation of the camera (extrinsic parameters).

The *Möller–Trumbore* ray-triangle intersection algorithm [24] is used to calculate the intersection between the ray and a triangle in 3D. Ray tracing computations that are performed between a gaze ray and all triangles of the 3D reconstructed space carry significant computational cost. To improve the 3D fixation localisation’s performance, we implement a pyramid model of the 3D reconstructed mesh. This consists of a triangulated model with N scaled triangles, and each of them consists of M triangles of the original model. The ray-triangles intersection algorithm is performed in two steps, between:

1. The ray and the N scaled triangles.
2. The ray and the M triangles included in the scaled triangle, which was intersected in step 1.

N and M are defined as:

5 $$\begin{aligned} \mathbf{N }= & {} \sqrt{{\varvec{2}}*{\varvec{n}}_{\mathbf{model }}} \end{aligned}$$

6 $$\begin{aligned} \mathbf{M }= & {} {\frac{{\varvec{n}}_{\mathbf{model }}}{\mathbf{N }}} \end{aligned}$$

where ${\varvec{n}}_{\mathbf{model }}$ is the number of the points of the 3D reconstructed mesh.

### Robotic laser task

**Fig. 5:**
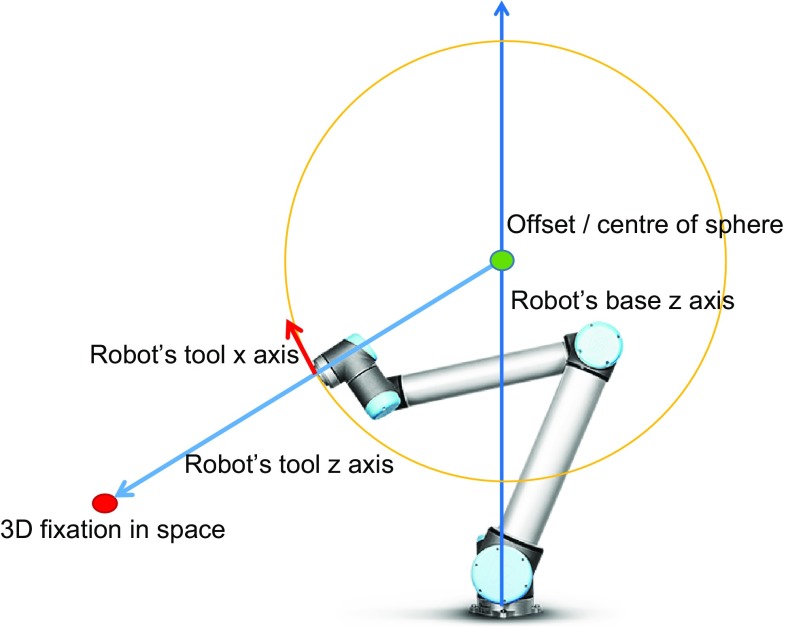
Estimation of robot’s pose to highlight the 3D fixation (sphere approach)

To highlight the 3D fixation in the operating room using a robotic laser, the estimated 3D fixation should be converted to a corresponding robot pose. To this end, the Kinect-to-Robot registration is not sufficient. We need to define one of the multiple poses with which the z-axis of the robot’s end-effector intersects the 3D fixation (Fig. 5). A sphere with a predefined radius is defined, and its centre is placed on a point along the z-axis of the robot. The intersection of the ray—defined by the coordinates of this centre point and the 3D fixation—with the sphere will be the translation of the robot’s end-effector. The rotation is defined by the z-axis of the robot’s end-effector, which should be aligned with the line defined by the sphere intersection and the 3D fixation. The x- and y-axes are set arbitrarily. Finally, the pose is transformed to the robot’s coordinate system and transmitted to it.

**Fig. 6:**
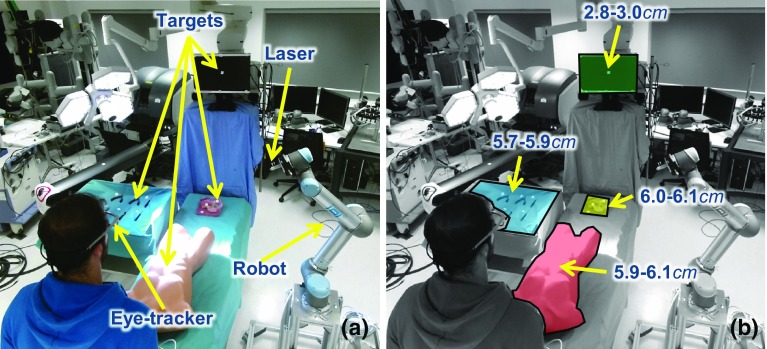
**a** The experimental set-up (view from the Kinect sensor): as the subject fixates on predefined targets, the pose of the eye-tracker scene camera is estimated. When a fixation is detected, the 2D gaze is mapped to 3D coordinates and the robotic laser highlights the fixated spot. **b** The error ranges within the main fixated areas of interest

## Experiments and results

For the experimental evaluation of the application presented here, 20 targets are placed in the operating theatre (Fig. 6, Online Resource 1). The distance range between the subject and the targets is 92–212 cm and between the robot and the targets 42–193 cm. The task involves fixating on the targets for more than 4  s, and this process is repeated 3 times. The accuracy and the real-time performance of the system are evaluated over 60 fixations. The real-time performance is evaluated based on the time interval between a user fixation being detected and the corresponding 3D fixation being calculated.

The accuracy of the system can be affected by multiple factors: the eye-tracker intrinsic error, the head pose estimation (ORB-SLAM) error, the robot calibration error and the Kinect sensor. In this validation we measure:

- The *eye-tracker error*, which is a 2D distance in pixels on the eye-tracker’s scene-camera frame, expressed as a % of its resolution (720p) and based on comparing the actual and the expected 2D fixations.
- The *framework error* comparing the actual and the expected 3D fixations (compounded by the eye-tracker’s error).
- The *robotic laser error* derived by the Kinect-to-Robot calibration and the laser module’s intrinsic offset**,** by manually repositioning the robot to accurately highlight the 3D targets.
- The *overall system error*, comparing 3D target coordinates with the 3D coordinates of the laser projection. This also depends on the geometry of the surface where the laser is projected.

**Fig. 7:**
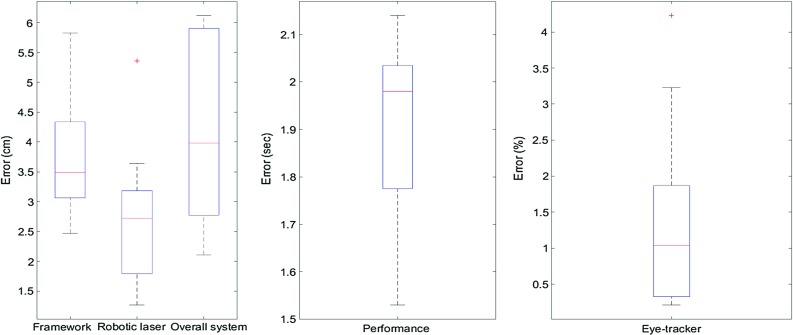
Error analysis represented in *box plots* generated by MATLAB software package. The median (*line in the box*), first and third quartiles (*box*), minimum/maximum values (*lines on top* and *bottom of the box*) and outliers (*cross*) are demonstrated

The results summarised in Fig. 7 show the median error, distribution, minimum/maximum values for the performance and the error over all measured fixations. The overall system’s median error is 3.98cm with 1.98-sec delay interval between the detection of the fixation and the activation of the robot.

**Fig. 8:**
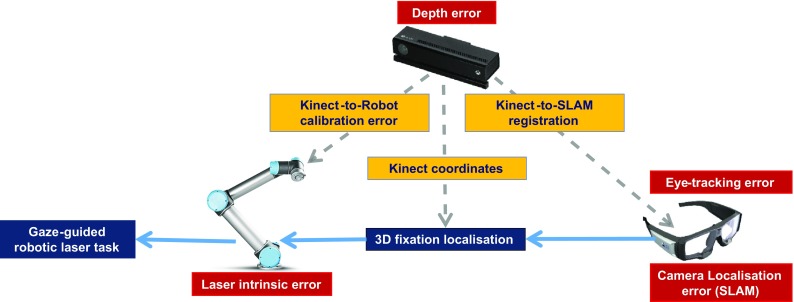
Sources of error and their interrelation. The Kinect sensor introduces a depth inaccuracy, which propagates to the system through its calibration with the robot, its registration with the SLAM local map and the 3D fixation localisation (in Kinect coordinates). Moreover, error is introduced and propagated towards the output of the system through the eye-tracker’s inaccurate gaze estimation, the inaccuracy of the ORB-SLAM algorithm, which localises the camera within the 3D space, and the error produced by the offset of the laser pointer (reduced after its calibration)

## Discussion and conclusions

An early implementation of a novel framework has been presented that allows gaze-driven interactions within a 3D environment and a collocated robotic manipulator. This is achieved by the combination of unrestricted wearable gaze tracking, theatre 3D reconstruction and advanced computer vision concepts. The experimental results presented here demonstrate an improvement of 31% on 3D fixation localisation accuracy compared to earlier implementations, while the overall system’s accuracy is 3.98 cm. Future work will focus on further reducing this error. Segmenting the 3D space and magnetising the fixation to objects of interest could further reduce the error. The current performance is 1.98 s for each 3D fixation localisation, which limits its use in applications that demand lower time intervals. This can be addressed with further software and GPU optimisation or statistical analysis of the user’s gaze behaviour. Another current limitation is the use of a static 3D model of the environment to allow near-real-time performance. This will be overcome by using point-based fusion [25] and other optimisations on a separate CPU. A dynamically updated 3D model will allow safety-critical tasks, such as real-time obstacle detection and robot path planning and occlusions avoidance of the laser projection.

It is of paramount importance to quantify the contribution of each constituent component of the implementation (hardware and methodologies) to the overall system error (Fig. 8). The Kinect sensor produces an average error of 2–4 mm, but depending on the distance from the target this may increase to over 4 mm [16]. This error propagates to multiple stages of the system and can be reduced using multiple Kinect sensors. Eye-tracking may introduce variable error due to the parallax effect [26] occurring over large fixation distances. This can be eliminated by performing multiple use-specific calibrations over multiple distances and then accordingly switch or interpolate between the derived calibration parameters based on a resolved fixation depth. The Kinect-to-Robot registration method used [20] exhibits an error of 0.75 cm for calibration using ${\sim }25$ poses. Automating calibration with more than 80 poses would reduce the error to ${\sim }0.5$ cm. Last but not least, the head pose estimation is one of the most significant stages of the framework and is speculated to introduce an error due to the complexity of the computer vision approach. Therefore, we aim to set up an optical tracking system to provide ground truth for clinical studies, further development of the SLAM-based approach and accurate validation.

It should be noted that the laser-holding robotic arm serves no clinical use case as presented. On this occasion the robot is used to demonstrate its integration and achieved accuracy by means of the presented application. Additionally, more economic ways are available for displaying one or more laser points in the theatre.

The work presented here represents an introduction and preliminary experimental validation of core functionalities of a larger framework under development. The proposed framework is geared towards a safer and more efficient surgical theatre. Applications, which aim at enhancing safety, collaboration and training, include: *gaze-guided object recognition and tracking, robotic manipulation, augmented visualisation of gaze relevant information, behavioural analysis and workflow segmentation based on perceptual information provided by the framework*. It is envisaged that an open-source and hardware-agnostic framework will allow large-scale deployment in several theatres. This would provide a large amount of easily anonymised data, which will help generate a large evidence base and critical mass for clinical use.

## Electronic supplementary material

Below is the link to the electronic supplementary material.

Video demonstrating the workflow of the experimental setup. The subject fixates on a target for more than 4sec and the robotic laser highlights this spot.(mp4 58.8MB)
